# Directional Decoding From EEG in a Center-Out Motor Imagery Task With Visual and Vibrotactile Guidance

**DOI:** 10.3389/fnhum.2021.687252

**Published:** 2021-09-24

**Authors:** Lea Hehenberger, Luka Batistic, Andreea I. Sburlea, Gernot R. Müller-Putz

**Affiliations:** ^1^Institute of Neural Engineering, Graz University of Technology, Graz, Austria; ^2^Laboratory for Application of Information Technologies, Faculty of Engineering, Department of Computer Engineering, University of Rijeka, Rijeka, Croatia; ^3^BioTechMed Graz, Graz, Austria

**Keywords:** vibrotactile guidance, kinesthetic guidance, motor imagery, electroencephalography, brain-computer interface, directional decoding

## Abstract

Motor imagery is a popular technique employed as a motor rehabilitation tool, or to control assistive devices to substitute lost motor function. In both said areas of application, artificial somatosensory input helps to mirror the sensorimotor loop by providing kinesthetic feedback or guidance in a more intuitive fashion than via visual input. In this work, we study directional and movement-related information in electroencephalographic signals acquired during a visually guided center-out motor imagery task in two conditions, i.e., with and without additional somatosensory input in the form of vibrotactile guidance. Imagined movements to the right and forward could be discriminated in low-frequency electroencephalographic amplitudes with group level peak accuracies of 70% with vibrotactile guidance, and 67% without vibrotactile guidance. The peak accuracies with and without vibrotactile guidance were not significantly different. Furthermore, the motor imagery could be classified against a resting baseline with group level accuracies between 76 and 83%, using either low-frequency amplitude features or μ and β power spectral features. On average, accuracies were higher with vibrotactile guidance, while this difference was only significant in the latter set of features. Our findings suggest that directional information in low-frequency electroencephalographic amplitudes is retained in the presence of vibrotactile guidance. Moreover, they hint at an enhancing effect on motor-related μ and β spectral features when vibrotactile guidance is provided.

## 1. Introduction

Injuries or diseases resulting in the loss or degradation of motor functions severely disrupt the lives of those afflicted by them in many ways. Therefore, researchers across several disciplines concern themselves with investigating methods to restore or replace lost functionality. On this quest, it is vital to consider the entire sensorimotor control loop, which contains both afferent and efferent processes. In natural movement processes, the feed-forward (i.e., execution of movement) and feedback (i.e., haptic information, proprioception, visual information etc.) processes cannot be viewed as decoupled. Rather, movement actions are adjusted and refined during the execution according to sensory inputs. Moreover, combining motor imagery (MI), where participants imagine performing movements without executing them, with afferent feedback has been shown to induce plasticity at the motor cortex level (Pichiorri et al., 2011, 2015; Mrachacz-Kersting et al., 2017b). In Mrachacz-Kersting et al. (2017b), a tight correlation has been observed between the afferent inflow caused by the electrical stimulation and electroencephalographic (EEG) motor-related patterns in the amplitude of low frequency bands, suggesting that both reach the somatosensory areas at similar times. Removing somatosensory feedback from natural movement decreases motor control, as documented for grasping movements with artificially removed haptic sensation (Johansson and Westling, 1984), and wrist movements with artificially disrupted proprioception (Galán et al., 2015). Furthermore, in the specific case of upper limb prostheses for amputees, surveys have found that many participants express a desire for tactile feedback (Biddiss and Chau, 2007; Pylatiuk et al., 2007; Lewis et al., 2012; Cordella et al., 2016), to make the interaction feel more natural.

The most common means to provide non-invasive somatosensory input are vibrotactile (Chatterjee et al., 2007; Cincotti et al., 2007; Antfolk et al., 2010; Leeb et al., 2013), electrotactile (Bach-y Rita and Kercel, 2003; Cincotti et al., 2012; Franceschi et al., 2016; Mrachacz-Kersting et al., 2017b; Corbet et al., 2018), mechanotactile (Patterson and Katz, 1992; Antfolk et al., 2013), or passive movement (Ramos-Murguialday et al., 2012, 2013; Mrachacz-Kersting et al., 2017b; Randazzo et al., 2017). These modalities are employed for different purposes, including force feedback (Patterson and Katz, 1992; Antfolk et al., 2010, 2013), transmission of kinesthetic information for proprioceptive (Ramos-Murguialday et al., 2013; Randazzo et al., 2017) or navigational purposes (Bach-y Rita and Kercel, 2003), or encoded patterns with discrete (Chatterjee et al., 2007; Cincotti et al., 2007), or continuous properties (Franceschi et al., 2016).

In studies or applications where there is no inherent (somatosensory) feedback, it is commonly substituted by visual input, since the visual modality offers a wide variety of possibilities, and the visual sense is capable of processing a large volume and variety of stimuli. In a context dealing with motor rehabilitation or replacement of motor function, many such works utilize motor imagery. Motor imagery may be subdivided into different modalities including visual MI (imagery of movement visualization) and kinesthetic MI (imagery of movement sensation) (Jeannerod et al., 1994). Neurophysiological comparison of kinesthetic MI and visual MI shows that kinesthetic MI yields more activity in motor-associated structures and inferior parietal lobule, while visual MI activates predominantly the occipital regions and the superior parietal lobules (Guillot et al., 2009; Chholak et al., 2019). Furthermore, kinesthetic MI and visual MI have been associated with event-related desynchronization (ERD) and event-related synchronization (ERS) (Pfurtscheller and Da Silva, 1999; Pfurtscheller and Neuper, 2001; Müller-Putz et al., 2005; Muller-Putz et al., 2006; Rohm et al., 2013), respectively, in said areas of the brain (Chholak et al., 2019). ERD and ERS are event-related phenomena that represent frequency-specific changes of the ongoing EEG activity and consist of local decrease or increase, respectively, of power in certain frequency bands (Pfurtscheller and Da Silva, 1999). When comparing classification accuracies of various movements, kinesthetic MI was proven to give better classification accuracy results than visual MI, while movement execution and observation of movement give better results than both visual MI and kinesthetic MI (Neuper et al., 2005). Furthermore, when inspecting classifier patterns, kinesthetic MI and movement execution had very similar areas of the brain with the most relevant electrode positions for the recognition of the respective task, specifically, being located above the central cortical area (Neuper et al., 2005).

Considering that movement processes suffer when somatosensory feedback is impaired (Johansson and Westling, 1984; Galán et al., 2015), it is increasingly believed that the performance of MI could benefit from artificial somatosensory input. To more closely simulate the motor control loop, in applications where MI has been employed as a control tool for end-effectors, a variety of different approaches have been studied to artificially provide somatosensory feedback. Somatosensory feedback or guidance in conjunction with MI tasks has principally been studied with respect to features derived from ERD. Studies comparing classification performance in MI tasks with either vibrotactile feedback or visual feedback (Cincotti et al., 2007; Leeb et al., 2013) have found no significant effects of the feedback modality. In Cincotti et al. (2007), most of the participants expressed that subjectively, the vibrotactile modality felt more natural. In a rehabilitation context, several works claim beneficial interaction effects between passive movement feedback, ERD elicited during MI, and motor scores. Specifically, Ang et al. (2009); Ramos-Murguialday et al. (2013) have documented improvements in Fugl-Meyer assessment motor scores in stroke patients following MI training with feedback in the form of passive joint movement. Ramos-Murguialday et al. (2012, 2013) further found that BCI performance of participants receiving passive movement feedback was higher compared to control groups which received sham feedback. Similarly, Randazzo et al. (2017) achieved a performance increase when adding passive movement guidance to an MI task, and Corbet et al. (2018) documented enhanced ERD during MI with electrotactile guidance, compared to visual guidance. While, Corbet et al. (2018) demonstrate that the electrotactile input does not directly produce ERD unless it exceeds the motor threshold, Hommelsen et al. (2017) found highly similar ERD patterns in the mu frequency band when comparing a motor task with sensory-threshold electrotactile feedback to sensory-threshold electrotactile stimulation without movement. We are not aware of any works documenting similar undesirable effects with vibrotactile input in the absence of a motor task. In a previous study by our group, we have found no ERD in a non-movement condition with vibrotactile sham feedback (Hehenberger et al., 2020). In this case, vibrotactile sham feedback was provided by “replaying” a feedback sequence from a previous trial.

Motor execution and motor imagery have been investigated by means of low frequency EEG amplitude during cue-based (Ofner et al., 2017; Schwarz et al., 2017) and self-paced tasks (Sburlea et al., 2015; Pereira et al., 2017, 2018). Movement-related cortical potentials (MRCPs) are neural specific patterns associated with the self-paced initiation of movement (Deecke et al., 1976). The pattern is characterized by a gradual negativity starting at ~1.5 s before the movement onset and reaching peak negativity in the proximity of the movement onset. According to Mrachacz-Kersting et al. (2017a), manual pressure stimulation produced beneficial effects on the MRCP variability in stroke patients performing a motor execution task. Furthermore, Mrachacz-Kersting et al. (2017b) found an increase in cortical excitability when healthy participants received either functional electrical stimulation or passive movement stimulation in response to the MRCP of imagined foot movements.

Several studies have shown that MRCPs also encode properties of the movement, such as speed, applied force (do Nascimento and Farina, 2008; Jochumsen et al., 2016) or directional information (Kobler et al., 2020a). Kobler et al. (2020a) has demonstrated that directional information is encoded around the low-frequency delta band. In their analyses of a pursuit tracking task, they found that at the beginning of the trials, directions were more discriminable when aligning the EEG to the time point where the target starts moving, than when aligning to the time point when subjects initiated the pursuit. Furthermore, they achieved better accuracies decoding directional information from parieto-occipital regions than from the sensorimotor areas. To our knowledge, the potential influence of tactile input on directional decoding from EEG has not been well-studied.

In a previous study by our group which involved executed center-out movements with real-time kinesthetic vibrotactile feedback (Hehenberger et al., 2020), we started to look into this issue tentatively. However, it was designed around a focus on movement-related correlates rather than directional decoding. Consequently, the results pertaining to directional decoding were largely inconclusive.

The present work focuses on directional decoding in a similar center-out motor task, while removing natural proprioception from the equation, in order to increase the salience of the vibrotactile input. The motor task has been modified to guided MI, and the vibrotactile input takes the form of vibrotactile guidance rather than feedback. In the following, we present the analysis of EEG signals recorded while participants performed this guided center-out MI task. We compare visual guidance against visual guidance supplemented by kinesthetic vibrotactile guidance, surmising that the presence of vibrotactile guidance maintains and potentially enhances performance for directional decoding, as well as the detection of a motor state.

## 2. Materials and Methods

### 2.1. Participants

The experiment was performed with 15 able-bodied participants (7 male, 8 female; age 21–32). All participants were self-reportedly right-handed. According to self-reports, 10 participants had prior experience with motor imagery, and six had prior experience with vibrotactile stimulation. At the beginning of the experiment, participants received both written and verbal instructions, before providing written informed consent. Participants received a monetary compensation of 7.50 € per hour for their efforts. The study protocol was approved by the ethical committee of the Medical University of Graz.

### 2.2. Experimental Setup

Participants were seated in front of a monitor, and instructed to perform guided motor imagery of a center-out arm movement task, i.e., slowly sliding their right palm across a flat surface. The imagery was guided by a visual moving cue displayed on the monitor, as well as a simultaneous vibrotactile moving sensation across the right shoulder blade (condition VtG: Vibrotactile Guidance), or by a visual moving cue alone (condition noVtG). The direction of the center-out movement was cued to one of two orthogonal directions: to the right, or up/forward. In the second case, the visual and vibrotactile guidance moved upwards, while the imagined movement was to the front. In the following, it will be referred to as “up.”

Several of the participants did not have prior experience with vibrotactile stimulation or motor imagery. Hence, participants were led through a familiarization procedure at the beginning of the experiment. This practice has also been suggested in Roc et al. (2020), in order to best prepare users to perform MI tasks. The familiarization included practice runs for each condition, and the option to practice executing the center-out movement on a table surface, in order to help them memorize the movement as vividly as possible.

**Figure 1:**
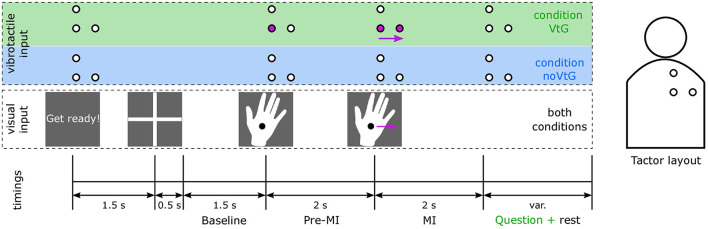
Trial structure according to the experimental paradigm, and tactor layout. The time intervals are indicated at the bottom. The middle row illustrates the visual cues, which were identical in both conditions, and the top row (highlighted in green and blue to identify the conditions VtG and noVtG, respectively) the vibrotactile guidance, respectively. The circles represent the tactors, as illustrated in the sketch of the tactor layout on the right. Active tactors are marked in purple, and idle tactors are presented in white. The purple arrows represent movement in both representations (visual and vibrotactile), in the presented example a movement to the right. The participant was visually alerted to the beginning of each trial, 1.5 s before the appearance of the fixation cross. The fixation cross was on screen for 2 s, the latter 1.5 s of which were later used as a baseline period. During this period, participants were instructed to fixate their gaze on the fixation cross, and relax. Afterwards, the visual cue, a right hand with a fixation point, appeared on the monitor. It remained stationary for the pre-MI period of 2 s, and then moved either to the right or up at a constant speed. Participants were instructed to perform the MI in accordance with the movement of the cue. In condition VtG, participants were subsequently asked to judge whether the vibrotactile guidance was congruent to the visual guidance in this trial, and to respond with a key press.

Following the familiarization, each participant completed three runs per condition, in a block design. The order of conditions was switched between participants, such that eight participants started with condition VtG and seven with condition noVtG, respectively. Runs consisted of 40 regular trials each, whereas in condition VtG, each run comprised four additional trials where the movement direction of the vibrotactile guidance and the visual guidance were incongruent. In condition VtG, participants were prompted to rate after every trial whether the two guidance modalities were congruent or not. Incongruent trials were used to help keep the participant engaged and were not used in the future classification and analysis. Each trial was 7.5 s long, where the MI task was performed for a period of 2 s, as shown in Figure 1.

After the main runs, two runs of continuous rest were recorded, with a duration of one minute each. Finally, participants were led through two runs of controlled eye artifacts, i.e., saccades and blinks (Kobler et al., 2020b).

The experimental paradigms were implemented on the simulation and neuroscience application (SNAP) platform (http://github.com/sccn/SNAP). SNAP builds on Python 2.7 and Panda 3D (https://www.panda3d.org).

After participating in the experiment, participants were asked to fill out a questionnaire which consisted of seven questions for condition VtG and five questions for condition noVtG. Questions were answered by selecting values from 1 to 5 on a Likert scale (1: Strongly disagree, 2: Disagree, 3: Neither agree nor disagree, 4: Agree, 5: Strongly agree). The questionnaire can be found in the Supplementary Material.

### 2.3. Vibrotactile Stimulation

Three specialized tactile actuators, i.e., C-2 tactors (Engineering Acoustics Inc., Casselberry, USA) were attached to the inside of an elastic shirt to stimulate the right shoulder blade. The tactors were controlled by a custom device containing an ARM Cortex M4 micro-controller (STMicroelectronics, Geneva, Switzerland). The carrier frequency of the signal driving the tactors was 250 Hz. The intensities of the C-2 tactors were manipulated in Python 2.7 via a serial interface. To control for individual sensitivity profiles, the tactor amplitudes were calibrated such that the perceived intensities were equalized. The vibrotactile guidance consisted of a moving virtual stimulus, which simulated a movement from the central tactor to one of the outer tactors. The tactor amplitudes to evoke the moving sensation were modulated according to the following mapping (Israr and Poupyrev, 2011; Luzhnica et al., 2017; Hehenberger et al., 2019, 2020),


(1) $$\begin{array}{l} x_{v}=\frac{A_{2}^{2}}{A_{1}^{2}+A_{2}^{2}} \end{array}$$

with *x*_*v*_ the location of the moving stimulus between the start tactor *T*_1_ (*x*_*v*_ = 0) and the end tactor *T*_2_ (*x*_*v*_ = 1), and *A*_1_, *A*_2_ the amplitudes of *T*_1_, *T*_2_, respectively.

### 2.4. Signal Acquisition

EEG and EOG was recorded from 64 actiCap electrodes using two BrainAmp amplifiers (Brain Products GmbH, Gilching, Germany), at a sampling rate of 1 kHz. Electrodes were arranged according to the international 10/10 EEG system (Chatrian et al., 1985), where 61 channels were used for EEG and three channels were used for EOG.

### 2.5. Signal Processing

We investigated two sets of features, i.e., low-frequency amplitude features around the δ range (0.2–5 Hz), and spectral power features, with a focus on the μ (8–12 Hz) and β (15–32 Hz) frequency bands. For both sets of features, we present neurophysiological characteristics, and classification results.

#### 2.5.1. Preprocessing

**Figure 2:**
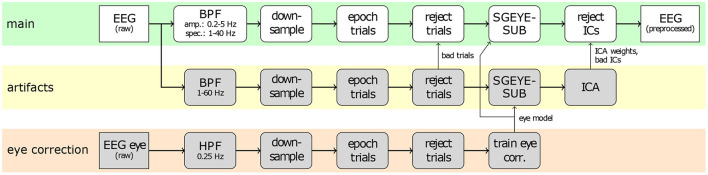
Common preprocessing of EEG signals. The top branch (highlighted in green) shows the main preprocessing applied to the trials, whereas the lower branches show the procedures used to correct for eye artifacts (bottom, orange), and general artifacts (middle, yellow).

Figure 2 provides an overview of the common preprocessing procedure. First, the raw EEG signals of the main runs were subjected to a feature-specific band-pass filter (4th order Butterworth, zero-phase), i.e., 0.2–5 Hz for low-frequency amplitude features, and 1–40 Hz for spectral features, respectively. The band-passed signals were downsampled to 200 Hz for neurophysiology analyses, as well as for classification based on spectral features, and to 10 Hz for classification based on low-frequency amplitude features. Subsequently, the signals were epoched into trials of 6 s, containing the baseline, pre-MI, and MI periods. In order to identify trials contaminated by artifacts, the raw signals were separately band-pass filtered between 1 and 60 Hz, and examined for their maximal amplitude, kurtosis, and joint probability. Trials with amplitudes over 200 μ V, or either the kurtosis or the joint probability exceeding five times the standard deviation were marked for rejection with the built in functions from EEGLAB toolbox (Delorme and Makeig, 2004; Delorme et al., 2007). On average, 20 trials per subject were rejected. Additionally, we employed the sparse generalized eye artifact subspace subtraction (SGEYESUB) algorithm (Kobler et al., 2020b) in order to attenuate eye movements correlated with the task. The SGEYESUB model was trained on the two runs of controlled eye artifacts. Finally, we performed an Independent Component Analysis (ICA) with the functionality provided by the EEGLAB toolbox. We used the Infomax algorithm (Bell and Sejnowski, 1995; Makeig et al., 1996) for the decomposition, and identified artifactual components based on SASICA (Chaumon et al., 2015), as well as on visual inspection of components. The ICA decomposition was performed on the signals filtered between 1 and 60 Hz, after the removal of contaminated trials, and the correction for eye movement artifacts, as described above. The ICA weights and rejected components were saved, and applied to the signals during the main preprocessing.

Afterwards, the signals were further subjected to specific processing depending on the type of analysis.

#### 2.5.2. Neurophysiology

In order to evaluate the low-frequency amplitude features, the preprocessed signals were re-referenced to the common average. For the spectral features, we performed a time-frequency decomposition using Morlet wavelets (Morlet et al., 1982) (FWHM = 3s @ 1 Hz), in 1 Hz steps from 1 to 40 Hz.

#### 2.5.3. Classification

We performed classification of three distinct aspects. First, we classified the two directions, separately for each condition, in order to assess how the vibrotactile guidance influences the directional discriminability. In this case, we considered the whole trial, producing a classification sample each 100 ms, where for each sample, the features were computed from the preceding 1 s of data. Second, we classified the MI period against the baseline, separately for each condition, to assess the impact of the vibrotactile guidance on the detection of a movement (MI) state. This classification was based on a fixed time window, i.e., 0.5–1.5 s after the cue movement onset for the MI, and 3.5–2.5 s before the cue movement onset for the baseline. Finally, we classified the two conditions against each other, considering the whole trial, as described above. For all classifications, we used a subset of 31 channels, covering frontocentral to parietal areas. We used linear discriminant analysis with shrinkage regularization (sLDA) for all classifications. All classifications were conducted two-fold, once using low-frequency amplitude features, and once using spectral features. Low-frequency amplitude features contained previous samples with a window size of 1 s (10 samples at 10 Hz). For classification based on spectral features, the preprocessed data were further filtered (4th order zero-phase Butterworth filter) in the two bands of interest, specifically μ (8–12 Hz) and β (15–32 Hz) frequency bands. To increase the separability of classes, common spatial patterns (CSP) (Ramoser et al., 2000; Blankertz et al., 2007; Ang et al., 2012) were calculated for each participant. CSP filters maximize the variance of the spatially projected signals for one class, while minimizing it for the other class. The CSP filters were calculated from a time window during the MI period, i.e., 0.5–1.5 s after the cue movement onset. The five most separating filters for each class were applied back on the data, giving 10 features. For each feature, the logarithmic power of each trial, relative to the baseline period, was calculated. This was done for μ and β frequency bands separately, and then the features from the two bands were combined. Finally, to obtain the classification features, a moving-average filter with a window size of 1 s was applied.

#### 2.5.4. Classification Feature Patterns

In order to identify spatial areas which most strongly contributed to the classification results, we used projections of the classification features into channel space.

For the amplitude features, we computed activation patterns, as suggested in Haufe et al. (2014):


(2) $$\begin{array}{l} a=\Sigma_{x}{\left(\Sigma_{x}+\lambda I\right)}^{-1}(\mu_{1}-\mu_{2})\cdot \;Var\{s\} \end{array}$$

with Σ_*x*_ the pooled covariance of the measurement signals **x**, λ the shrinkage parameter, μ_1_, μ_2_ the classwise means, and **s** the source estimate. The source estimate results from the backward model, i.e., the sLDA weights **W** applied to the measurements **x**:


(3) $$\begin{array}{l} \text{s}={\text{W}}^{T}\text{x} \end{array}$$

Since the spectral features were transformed via CSP, they cannot directly be shown in topographic plots. For this reason, instead of the classifier patterns, we present the CSP features projected to the channel space. In order to obtain CSP features, we computed the CSP model **M** (where each row is a CSP filter) and applied it on our data **x**:


(4) $$\begin{array}{l} \text{F}=\text{M}\text{x} \end{array}$$

After this, we calculated the pseudo-inverse of the CSP model **M** and applied it to the CSP features **F**, in order to project the data back to the channel space:


(5) $$\begin{array}{l} F_{ch}=M^{+}F \end{array}$$

From there, the logarithmic power of the **F**_*ch*_ (relative to the baseline period) was calculated for both classes. The difference of the powers between two classes was then calculated and later shown in topographic plots as power feature CSPs **P**:


(6) $$\begin{array}{l} \text{P}={\text{P}}_{1}-{\text{P}}_{2} \end{array}$$

#### 2.5.5. Statistics

We evaluated the performance of the classifications by computing a threshold of significantly better-than-chance accuracies, according to Müller-Putz et al. (2008). Furthermore, we conducted Wilcoxon signed-rank tests on the peak accuracies of the direction classification, as well as on the accuracies resulting from the classification between baseline and the MI period, to evaluate potential effects in performance between the two conditions.

## 3. Results

### 3.1. Neurophysiology

#### 3.1.1. Potentials

**Figure 3:**
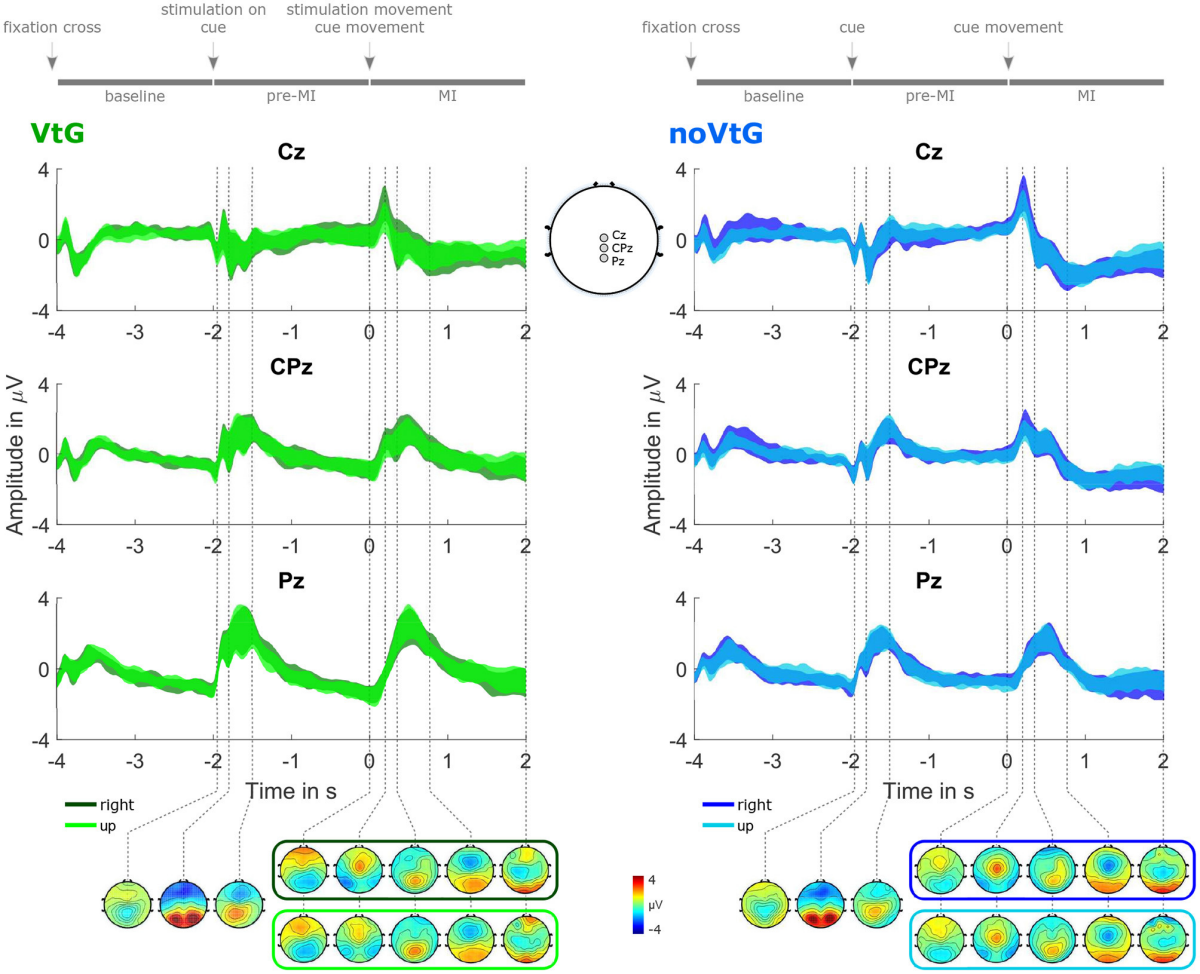
Grand-average potentials (0.2–5 Hz), for each condition, and direction. The potentials are represented as 95% confidence intervals of the amplitudes at Cz, CPz, and Pz, complemented by topographic plots at selected time points. The left panel depicts the potentials for condition VtG, whereas the dark green trace corresponds to the direction right, and the light green trace to the direction up, respectively. Similarly, in the left panel, the potentials for direction right are shown in dark blue, and the potentials for direction up in light blue. For time points before the cue movement onset, one set of topographic plots per condition is presented, while for time points after the cue movement onset, one set per condition and direction is presented. These plots are framed with a color-coded frame matching the color of the amplitude traces of the corresponding direction. The sketch in the middle at the bottom highlights the electrode positions whose amplitude traces are shown at the top.

Figure 3 illustrates the potentials between 0.2 and 5 Hz during an average trial. The potentials are presented separately for each condition, and direction, as 95% confidence intervals of the amplitudes at channels Cz, CPz, and Pz, and as topographic plots at selected time points.

There are prominent evoked responses to the stimuli provided by the paradigm, as shown in Figure 3. Visual evoked potentials (VEPs) are present in both conditions, following the appearance of the fixation cross, the appearance of the visual cue, as well as the start of the cue movement, whereas the VEP evoked by the fixation cross is smaller than the other two. In condition VtG, the second VEP overlaps with a somatosensory evoked potential (SEP) elicited by the onset of the vibrotactile stimulation. During the MI period, we can observe an MRCP presenting as a central negativity peaking within a second after the cue movement onset, which partially overlaps with the VEP. The peak amplitudes, slopes, and spatial profiles of the VEP and the MRCP slightly vary with the movement direction, and the condition. The MRCP negativity is stronger in condition noVtG (peak mean ± std at *t* = 0.77 s: −0.60 ± 1.71 μV in VtG, −1.83 ± 1.79 μV in noVtG), yet spatially broader in condition VtG. In both conditions, it is initially located precentrally, and later more centrally, with the later component exhibiting a contralateralization in condition VtG, but not in condition noVtG.

#### 3.1.2. Time-Frequency Analysis

**Figure 4:**
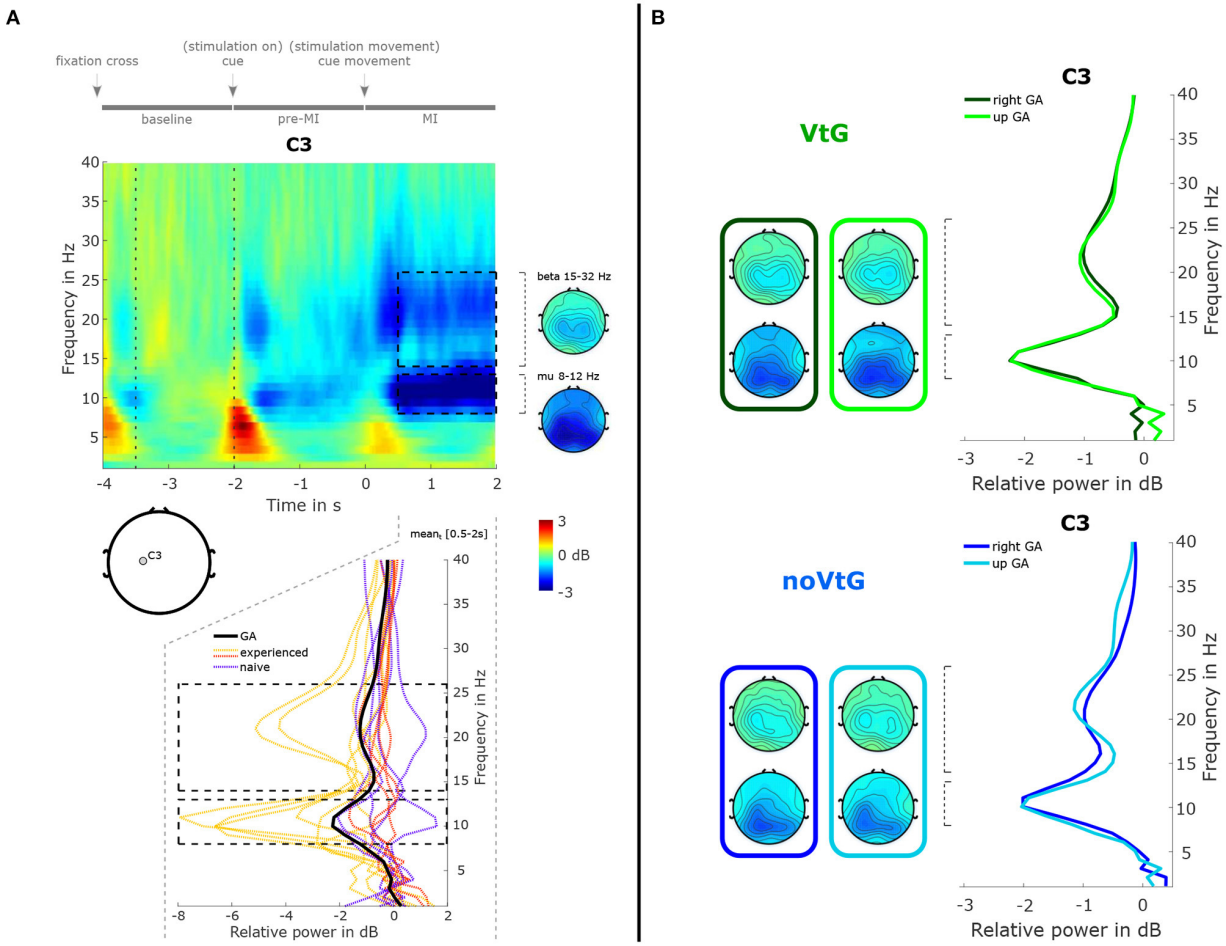
Summary of spectral features. **(A)** Time-frequency decomposition of the grand-average trial, as time-frequency map, topographic plots, and power spectrum. The time-frequency decomposition and spectrum are computed relative to the period marked by dotted vertical lines in the time-frequency map. The time-frequency map and spectrum are depicted at location C3. The topographic plots were computed from the ranges marked by the black dashed lines in the time-frequency map, i.e., 0.5–2 s in the time dimension, and 8–12 Hz for the μ band, and 15–32 Hz for the β band in the frequency dimension. The bottom panel depicts the grand-average (black solid line) and single-subject spectra (colored dashed lines), respectively, during the MI period (0.5–2 s). The single-subject spectra are grouped into three subgroups, i.e., MI-experienced with stronger than average spectral peaks (yellow), MI-experienced with average or weaker μ peaks (red), and MI-naïve (purple). **(B)** Power spectra at C3 and topographic plots for the two conditions, and two directions. Green and blue colors identify the conditions. Spectra and topographic plots were computed from the same ranges marked in **(A)**.

Figure 4A presents a time-frequency map of the grand-average trial (both conditions), along with topographic plots of the μ and β bands, and the relative power spectrum for the MI period. The spectrum plot includes the grand-average, and the individual power spectra.

Here, we can observe a power decrease in the μ and β frequency ranges during the MI period over centro-parietal areas, as well as a weaker decrease during the pre-MI period. As is evident from the individual relative power spectra in Figure 4A (bottom panel), there is considerable inter-subject variance. In the group of participants with prior MI experience, six individuals showed stronger-than-average desynchronization (yellow, μ peaks mean ± std: −5.52 ± 2.18 dB), and four showed weaker-than-average desynchronization (red, μ peaks mean ± std: −1.26 ± 1.00 dB). In the group of participants with no prior MI experience, all five individuals exhibited average or weaker-than-average desynchronization (purple, μ peaks mean ± std: −1.24 ± 0.92 dB). In Figure 4B, the grand-average spectra are depicted separately for each condition, and each direction. The spectral profiles are highly similar between the conditions and directions, while the μ peak is slightly stronger in condition VtG (−2.25 vs. −2.04 dB). Topographically, the strongest decrease is located over centro-parietal areas in both conditions, whereas in noVtG, they are more lateralized to the contralateral hemisphere.

### 3.2. Classification Results

#### 3.2.1. Directions

**Figure 5:**
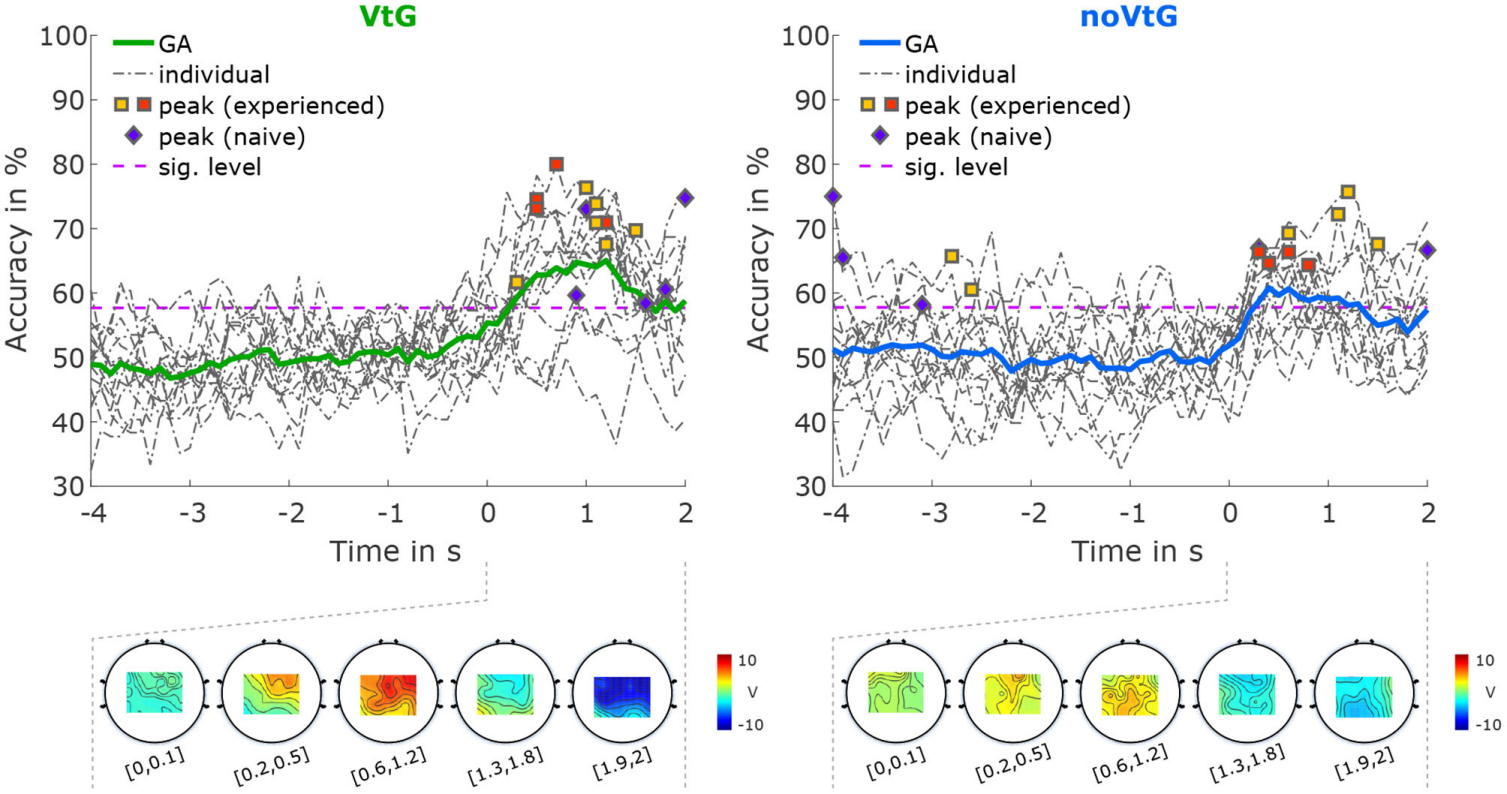
Classification results for direction right vs. direction up, based on amplitude features. The grand-average accuracies are depicted as thick solid lines, in green for condition VtG, and in blue for condition noVtG. Single-subject accuracies are represented by dash-dotted dark gray lines, and the individual peak accuracies are marked with squares for MI experienced participants, and diamonds for MI naïve participants, with the same color coding as in Figure 4. Below the accuracy plots, the activation patterns are presented for selected time intervals during the MI period.

The accuracies for classification between directions based on amplitude features are depicted in Figure 5. The average peak accuracy, which is obtained by averaging the subject-specific peak accuracies, is 69.67% in condition VtG, and 67.01% in condition noVtG, respectively. The subject-specific peak accuracies are not significantly different between the two conditions, as per a Wilcoxon signed-rank test (*p* = 0.1354). In both conditions, the activation patterns are strongest between 0.6 and 1.2 s after the cue-movement onset (positive), where the accuracies of most individuals reach their peaks, and at the end of the trial (negative). Spatially, the activation is strongest in central and frontocentreal areas in condition VtG, while in condition noVtG, it is concentrated over central and parietal areas.

Classification between directions based on spectral features did not yield accuracies significantly exceeding chance level. Therefore, the accuracies are not displayed here, but are added to the Supplementary Materials.

#### 3.2.2. Motor Imagery vs. Baseline

**Figure 6:**
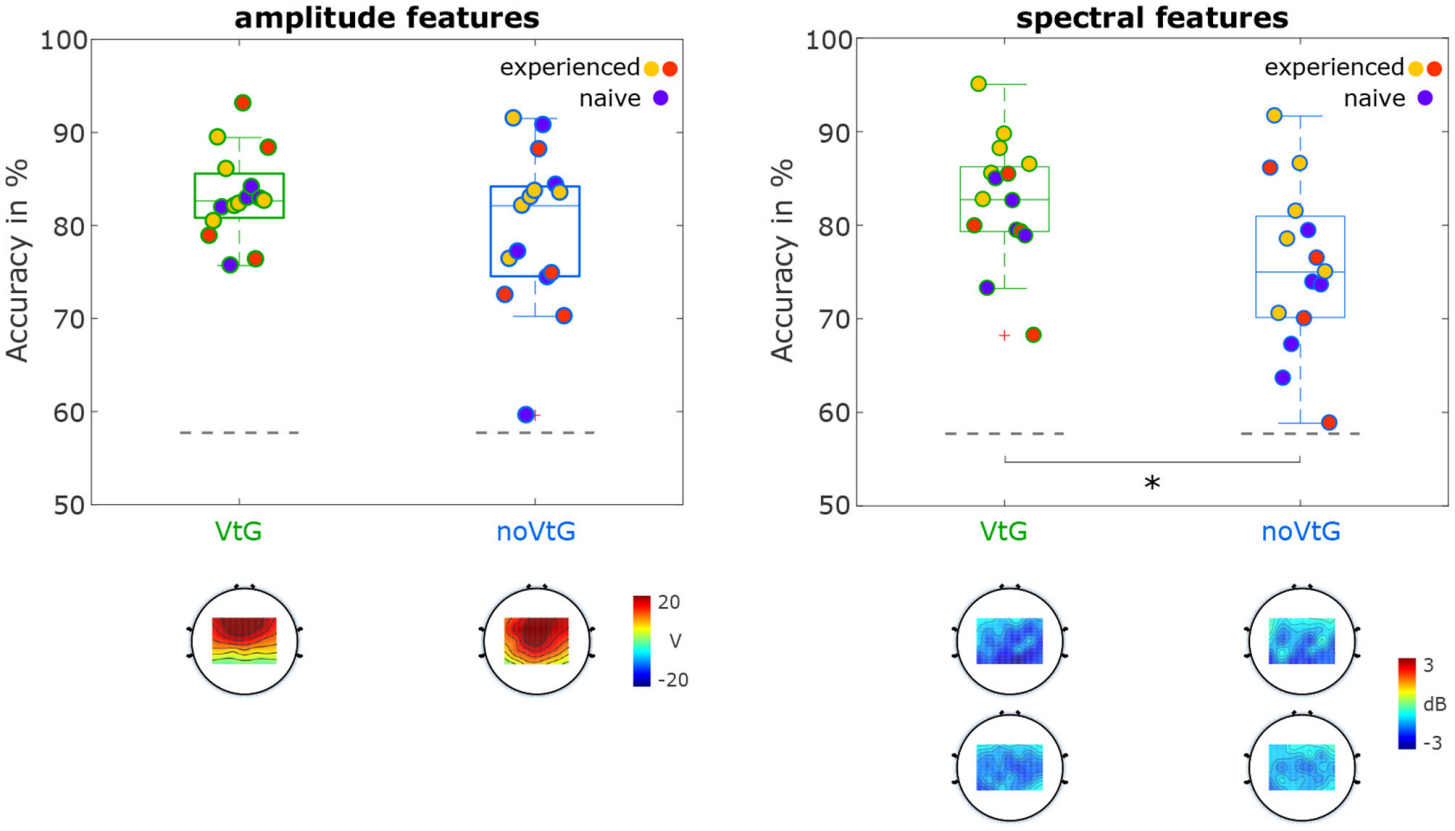
Classification results for MI vs. baseline. The distributions of accuracies are presented as box plots, with the individual accuracies identified with the same color coding as in Figures 4, 5. Statistical difference is marked with an asterisk. The activation patterns of the amplitude features, and the power feature CSPs are shown below the box plots.

Figure 6 depicts the distribution of accuracies for classification of MI against baseline, separated by condition. In both conditions and for both amplitude and spectral features, the maximal accuracies are over 90%, while the lowest accuracies range from 58.8% (condition noVtG, spectral features) to 75.7% (condition VtG, amplitude features). For both sets of features, the average accuracies are higher in condition VtG (amplitude 83.2%, spectral 82.6%) than in condition noVtG (amplitude 79.5%, spectral 75.5%), and the variances of the individual accuracies are lower in condition VtG. For the spectral features, the signed-rank test revealed a significant difference in the individual accuracies (*p* = 0.0012), while for the amplitude features, the difference is not significant (*p* = 0.0730). The result of the significance tests parallels the difference in the medians, which is marked in the box plots. For the spectral features, the median in condition VtG (82.7%) is higher than in condition noVtG (75.0%), whereas for the amplitude features, they are virtually identical (VtG 82.6%, noVtG 82.1%). The activation patterns for the amplitude features are concentrated in central channels in condition noVtG, and a little more frontal in condition VtG, with comparable intensity. The power feature CSPs are concentrated in parietal and central areas and are stronger in the μ frequency band than in β frequency band. Spatially, the patterns are highly similar in both conditions, whereas they are slightly stronger in condition VtG.

#### 3.2.3. Conditions

**Figure 7:**
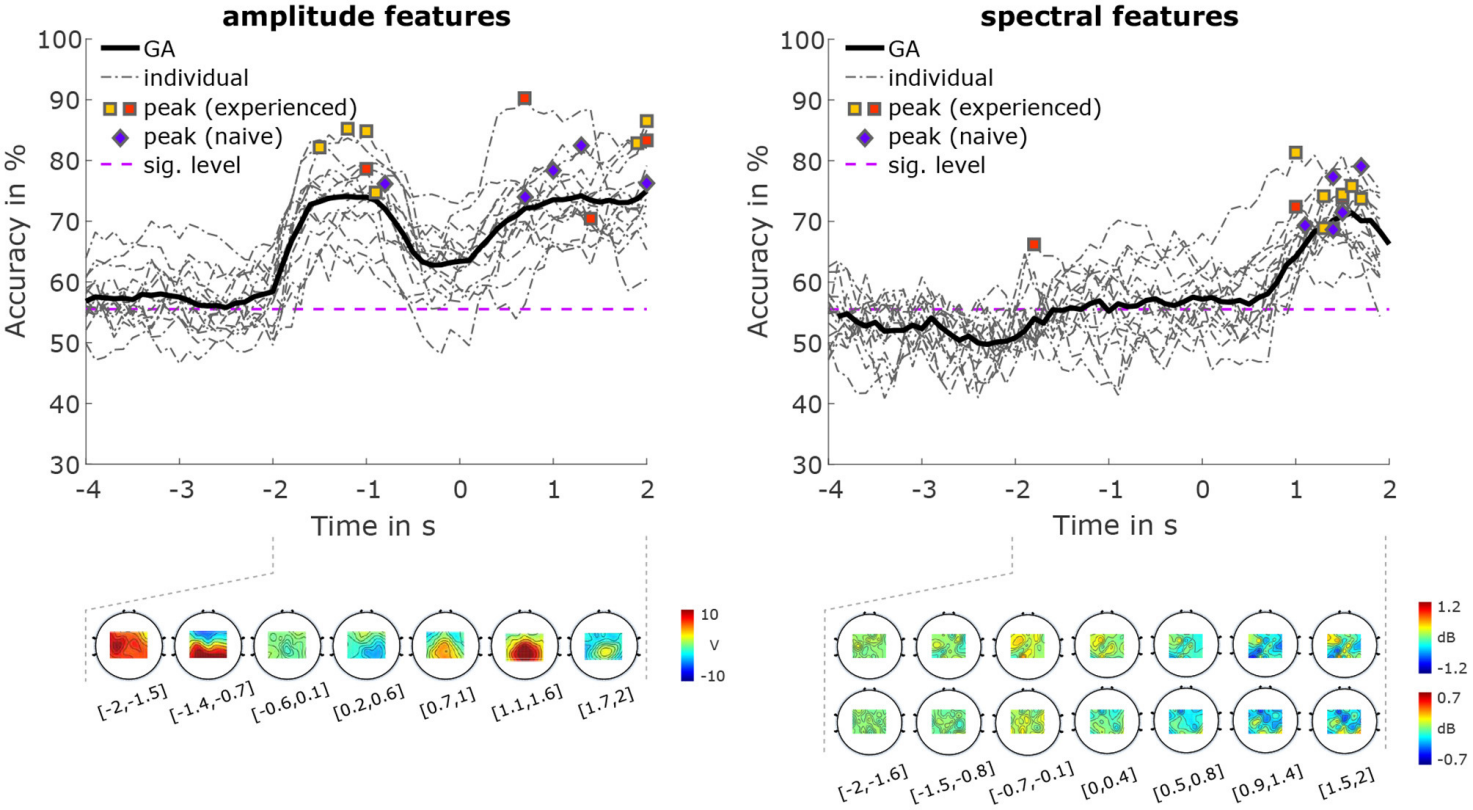
Classification results for condition VtG vs. condition noVtG, for amplitude features **(left)**, and spectral features **(right)**. The grand-average accuracies are depicted as thick black lines, single-subject accuracies as dash-dotted dark gray lines. The individual peak accuracies are marked with squares for MI experienced participants, and diamonds for MI naïve participants, with the same color coding as in Figures 4–6. Below the accuracy plots, the activation patterns of the amplitude features (bottom left), and the power feature CSPs of the spectral features (bottom right) are presented for selected time intervals within the pre-MI and MI periods.

The classification accuracies for classifications between the two conditions are shown in Figure 7. Using amplitude features, the average peak accuracy was 80.39% (SD 5.41%). Accuracies of over 70% were achieved both during the MI period, and the pre-MI period, with a maximum grand average accuracy of 75.00%. For spectral features, we achieved an average peak accuracy of 73.04% (SD 4.25%), and a maximum grand average accuracy of 71.4%. The activation pattern of the amplitude features during the pre-MI period is most pronounced between −1.4 and −0.7 s. This pattern is strongly positive in parietal areas, and negative in frontal areas. During the MI period, the patterns are strongly positive in central areas, peaking between 1.1 and 1.6 s. power feature CSPs (Figure 7) for the μ frequency band show that during the MI period (specifically, from 0.5 to 2 s), there is more negativity (i.e., a greater power decrease in condition VtG than in condition noVtG) in parietal areas. During the same time period, we can see more positivity (i.e., a greater decrease in power of condition noVtG than in condition VtG) in frontal areas. During the pre-MI period (specifically, from −0.7 to 0.4 s), there is a positivity (i.e., a greater decrease in power of condition noVtG than in condition VtG) in frontal areas. Power feature CSPs for the β frequency band during the MI period (specifically, from 0.5 to 2 s) are is a more negative (i.e., a greater power decrease in condition VtG than in condition noVtG) in central motor areas, and more positive in posterior parietal areas. During the pre-MI period (specifically, from *t* = −0.7 to *t* = 0.1*s*), there is a positivity (i.e., a greater decrease in power of condition noVtG than in condition VtG) in central and parietal areas.

### 3.3. Behavioral Results

**Figure 8:**
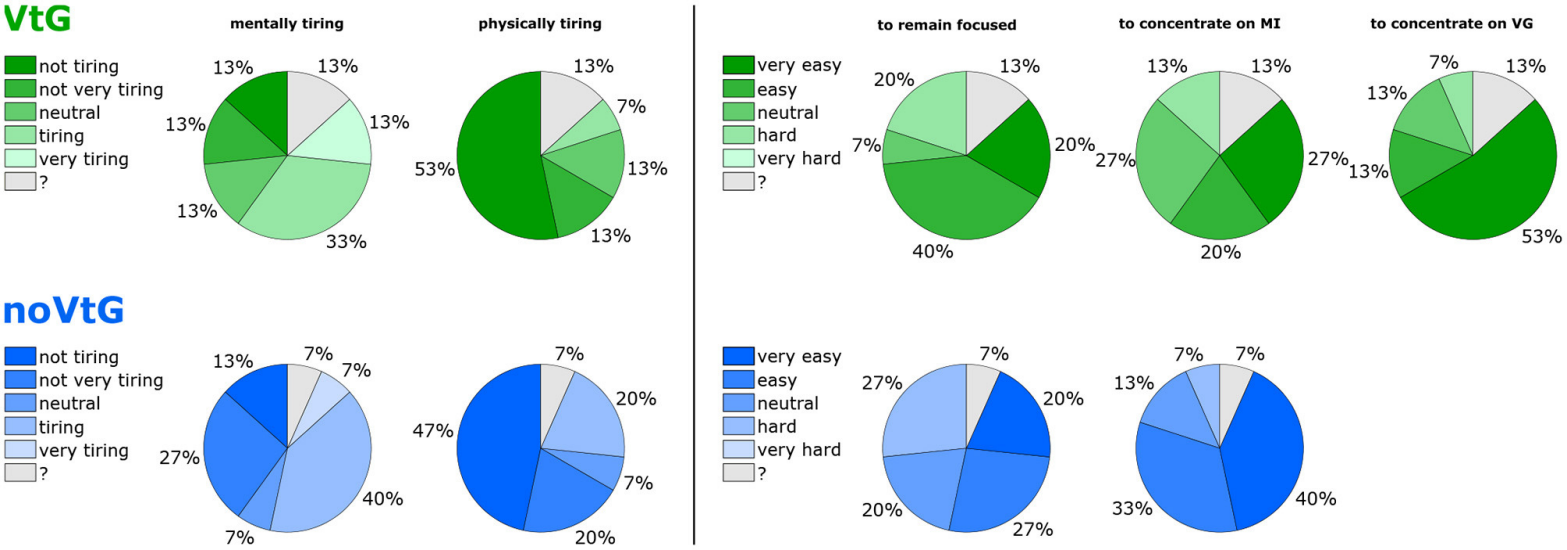
Overview of subjective ratings on the questionnaire. The **(top)** row (green colors) refers to condition VtG, the **(bottom)** row (blue colors) to condition noVtG. Darker colors correspond to a lower rated effort (e.g., “not at all tiring,” or “very easy to concentrate.” The gray portions identify invalid/missing answers.

Figure 8 shows a summary of participants' answers to selected questions on the questionnaire. Here, darker shades of green and blue correspond to a lower rated effort, and lighter shades to a higher rated effort. Two participants did not fill out the questionnaire correctly (one fully, one partly). The missing answers are indicated by question marks (gray segments).

On average, each condition was rated close to neutral with respect to mental tiresomeness (average ratings: VtG 3.2, noVtG 3.0). Three participants individually found condition VtG more tiring than condition noVtG, while one participant found condition noVtG more tiring. With respect to physical tiresomeness, both conditions were rated as “not very tiring” on a group level (average ratings: VtG 1.7, noVtG 2.0), where one individual found condition noVtG more tiring, and none found VtG more tiring. No participant gave a rating of 5 in this category for either condition.

For condition VtG, participants on average stated that they found it easy to remain focused throughout the experiment, and to concentrate on the MI task (average ratings: 3.7 in both cases). Furthermore, they found it easy to concentrate on the vibrotactile guidance (average rating: 4.3). For condition noVtG, they judged it as slightly harder to remain focused (average rating: 3.4), yet slightly easier to concentrate on the MI task (average rating: 4.1). Individually, two participants found it easier to remain focused in condition VtG, and one participant in condition noVtG, respectively. Three participants found it easier to concentrate on the MI task in condition VtG, and five vice versa.

Furthermore, all participants were confident or very confident that they detected the majority of the incongruent trials. On average, participants correctly detected 10.07 (SD 1.94) of 12 incongruent trials, and wrongly detected 0.63 (SD 1.25) of 120 regular (congruent) trials as incongruent trials. More detailed statistics of the detection of incongruent trials can be found in the Supplementary Materials.

## 4. Discussion

We have studied amplitude and spectral features in EEG signals recorded during a center-out MI task guided by a visual cue in conjunction with kinesthetic vibrotactile guidance (condition VtG), or by a visual cue alone (condition noVtG), focussing on directional and movement-related information. When classifying between directions for amplitude features, we found better average accuracies, as well as stronger activation patterns in condition VtG, but the peak accuracies were not significantly different. Classification of MI against baseline gave good accuracies in either condition, for both amplitude and spectral features. Mean accuracies were higher in condition VtG (significantly so for spectral features, where power feature CSPs were stronger than in condition noVtG). These results confirm that the directional and movement-related information are not adversely affected by the vibrotactile guidance. An enhancement could only be substantiated with respect to the motor state detection from spectral features.

Most participants performed well in detecting which trials were incongruent, i.e., 11 of 15 participants correctly detected 75% or more incongruent trials. We did not find a link between the rate of correctly detected incongruent trials, and the performance in the presented analyses.

### 4.1. Neurophysiology

The grand-average potentials shown in Figure 3 exhibit slight differences in the positive peak amplitude of the EP evoked by the cue movement onset between directions, where the positive peak is stronger in right trials, in both conditions. Possibly, the inherent internal mapping between the cue moving up and the imagined movement to the front may influence the intensity of the potential. The positive peak is followed by a combination of two negative peaks, the first of which we attribute to the late negative component of the evoked potential, while the second one vaguely presents the characteristics of an MRCP as a central negativity, which is expected when the imagined movement is initiated. Since the onset of the MRCP depends on when exactly the imagery is initiated, and thus varies between subjects and between individual trials, it is unsurprising that there is considerable variance in the grand-average potential. The spatial profile of the MRCP seems to be influenced by the vibrotactile guidance, since it is more contralateral in condition VtG.

Regarding the μ and β power decrease during the MI period illustrated in Figure 4, there is considerable inter-subject variability with three individuals exhibiting particularly strong patterns, and five individuals with very weak or uncharacteristic patterns. Part of this variability may be explained by the level of experience with MI tasks. In fact, all of the six participants with better-than-average μ and β peaks possessed prior MI experience, and the five MI naïve participants are among the eight weakest mu/beta peaks. However, it is well documented that ERD/ERS profiles can generally vary considerably between individuals (Allison and Neuper, 2010; Blankertz et al., 2010; Ahn and Jun, 2015; Wriessnegger et al., 2020), and while MI is a skill that can be trained and refined, naïve subjects may exhibit weak or strong patterns as a result of their neuroanatomy. The separation in the strength of the power profiles between the three groups highlighted in Figure 4 is to some extent also visible in the classification results of MI against baseline based on spectral features in Figure 6, especially in condition VtG. Between conditions and directions, the grand average spectral profiles show small variations. The μ peaks are fairly consistent, especially in experienced subjects, and the β peaks vary slightly in strength, where the peaks for direction up are stronger. Spatially, the pattern in condition VtG is more bilateral, compared to condition noVtG, where it is mostly contralateral. Furthermore, the pattern is slightly broader in condition VtG.

It is worth mentioning that condition VtG contained an additional task, i.e., responding after every trial whether the two guidance modalities were congruent, as mentioned in section 2.2. However, this task was executed outside of the main trial period, and with enough temporal distance to avoid contamination of the signals of interest by the additional motor activity of the key press motion. On average, the key press occurred 2.4 s after the end of the trial, and afterwards, ~5–6 s passed before the start of the baseline of the next trial. To our best knowledge, these offsets are sufficient to avoid any overlap of the signals of interest with ERD (Pfurtscheller and Da Silva, 1999) or an MRCP (Deecke et al., 1976) elicited by the key press movement.

### 4.2. Classification

For classification between directions based on amplitude features (Figure 5), accuracies exceeded the significance threshold during the MI period, with peak accuracies of 58–80% (grand average 65%) in condition VtG, and 58–76% (grand average 61%) in condition noVtG. While the difference in peak accuracies is not significant, the accuracies in condition VtG show less inter-subject variability. Furthermore, condition VtG exhibits stronger activation patterns, which appear as a mixture of central and parietal activations. The activation patterns in condition noVtG, on the other hand, are more centered in postcentral and parietal areas, but strongest parietally. Kobler et al. (2020a) found that directional information is encoded both in parietal areas, and to a lesser degree in central motor areas. Evidently, the classification results presented here are a product of a combination of both networks, whereas motor areas seem to be more strongly represented in condition VtG. It is worth pointing out that Kobler et al. (2020a) concluded that the decoding performance is not driven by differences in EPs.

When classifying the MI periods against the baselines (Figure 6), the best performing individual in each condition and for each set of features achieved accuracies exceeding 90%. For both sets of features, better results, i.e., higher mean, and lower variance of accuracies, were achieved in condition VtG, whereas the medians are only significantly different for spectral features. The power feature CSPs show that in both μ and β frequency bands, there is a decrease in power during the MI period, relative to the baseline, in parietal and central areas in both conditions. In both conditions, the decrease is stronger in the μ frequency band. The decrease is stronger (in both frequency bands) in condition VtG than in condition noVtG, which indicates that the vibrotactile guidance has more impact on the power difference between MI and baseline, and specifically, the biggest impact is in the μ frequency band. The activation patterns of the amplitude features, on the other hand, differ slightly in their spatial distribution, where the pattern for condition VtG is concentrated more frontally. The patterns reflect the spatial profiles of the potentials within the time window used for these classifications.

Classification between conditions (Figure 7) revealed that the signals are well-discriminable during the MI period, with both sets of features achieving peak grand average accuracies above 70%. For amplitude features, a comparable performance is reached during the pre-MI period, where in some participants, the peak accuracy during the pre-MI period is even higher than during the MI period. Considering the activation patterns, however, the discriminability in these two intervals is based on different underlying discrepancies. While during the MI period, the pattern is focused on central and postcentral areas, the pattern during the pre-MI period is composed of a positive parietal activation, and a negative frontal activation. For spectral features, on the other hand, the accuracy during the pre-MI period only slightly increases compared to the baseline, and hovers very narrowly above the significance threshold (peak GA 57%). This suggests that the vibrotactile stimulation does not per se induce ERD without the presence of a motor task, reinforcing an earlier finding from Hehenberger et al. (2020) cited in the introduction. There, no ERD was found in a non-movement condition with vibrotactile stimulation. Power feature CSPs show that in both μ and β frequency bands, there is a decrease in power during the MI period. In the μ band it is more parietal, while in the β band it is more central. Since changes in power happen at the same time (during MI) for both frequency bands, but are on different topological areas, this could explain why the best classification results were achieved when combining both frequency bands, and indicate that both μ and β bands harbor important information about change in power for our feature classification.

### 4.3. Behavioral Results

According to the ratings provided on the questionnaire, participants were split on how mentally tiring they perceived the task. In both conditions, a slim plurality leaned toward higher ratings (more tiring). On the other hand, most participants gave low ratings on how physically tiring it was, and none rated it as very physically tiring. In these two categories, the differences between the two conditions were relatively small. In fact, all but four participants gave the same rating in both conditions. Regarding the questions whether they were able to remain focused, and whether it was easy to concentrate on the motor imagery task, and on the vibrotactile guidance, respectively, no participant gave the lowest rating, i.e., none found it very hard to remain focused, or to concentrate on the task or the guidance. Interestingly, more participants responded that they were well able to remain focused during condition VtG, while more participants responded that they found it easy to concentrate on the motor imagery task in condition noVtG.

The aggregate of these results lead us to surmise that the influence of the vibrotactile guidance on these markers is predominantly subjective. This would correspond with informal feedback we have received from participants in previous studies with vibrotactile stimulation, and in pilot tests for this study. While our results on this aspect are mixed, Corbet et al. (2018) did find that the workload (specifically, frustration, effort, and mental demand) of an MI task was significantly lower with electrotactile guidance than with visual guidance. Furthermore, Cincotti et al. (2007) reported in a series of studies comparing vibrotactile feedback to visual feedback to an MI task that most participants found the vibrotactile feedback to feel more natural.

It should be noted that the behavioral data was collected at the end of the experiment, where subjective impressions of the first condition might not be fully accurate. Since the behavioral results were not part of our main hypothesis, we opted to collect these data jointly at the end of the experiment. For a more thorough analysis of behavioral data, it would be advantageous to collect data more frequently (e.g., after each condition).

## 5. Conclusion

The two orthogonal center-out movement directions were discriminable in low-frequency EEG amplitudes with moderate accuracies in both conditions, significantly exceeding chance level. The average performance is slightly higher when the vibrotactile guidance was present, though the individual peak accuracies do not differ significantly. Furthermore, we achieved moderate to decent accuracies (up to 95%) when classifying the MI period against the baseline, using either low-frequency amplitude features, or μ and β band spectral features. Average accuracies were higher, and less variable in condition VtG, though this improvement is only significant for spectral features. Based on these findings, we conclude that the vibrotactile guidance does not impede either the extraction of directional information or the detection of motor imagery, and perhaps provides beneficial effects in both cases. Therefore, we see vibrotactile guidance as a viable option to feasibly supplement visual guidance, while in applied cases, individual preferences should be taken into account.

## Data Availability Statement

The raw data supporting the conclusions of this article will be made available by the authors, without undue reservation.

## Ethics Statement

The studies involving human participants were reviewed and approved by Ethical Review Board of Medical University Graz. The patients/participants provided their written informed consent to participate in this study.

## Author Contributions

LH, AS, and GM-P had the idea. LH and LB performed the data recording, data analysis, and made the figures. LH, LB, and AS performed the paradigm design or implementation, wrote the initial draft, and wrote the manuscript. LH, LB, AS, and GM-P performed the proof-reading and editing. All authors contributed to the article and approved the submitted version.

## Funding

This work was supported by the ERC Consolidator Grant no. 681231 Feel Your Reach.

## Conflict of Interest

The authors declare that the research was conducted in the absence of any commercial or financial relationships that could be construed as a potential conflict of interest.

## Publisher's Note

All claims expressed in this article are solely those of the authors and do not necessarily represent those of their affiliated organizations, or those of the publisher, the editors and the reviewers. Any product that may be evaluated in this article, or claim that may be made by its manufacturer, is not guaranteed or endorsed by the publisher.
