# Supplementary material for: Directional Decoding From EEG in a Center-Out Motor Imagery Task With Visual and Vibrotactile Guidance
Source: Front Hum Neurosci. 2021 Sep 24;15:687252. doi: 10.3389/fnhum.2021.687252 (PMC8497713; doi:10.3389/fnhum.2021.687252)
Supplement: Supplementary file 1 [file Data_Sheet_1.PDF]

EXPERIMENT QUESTIONNAIRE (kfb2)

- (filled out by experiment conductor) Subject code: ________ Date: ________________
- Age: ________
- Sex (M/F): ________
- Left-handed or right-handed (L/R): ________
- Prior to this experiment, I participated in some sort of EEG Motor Imagery experiment.

YES NO

Following questions are related to the condition of the experiment in which **vibrotactile guidance was not present**. Please, read the questions carefully and answer each question by circling one number from 1 to 5. (1: Strongly disagree, 2: Disagree, 3: Neither agree nor disagree, 4: Agree, 5: Strongly agree)

1. Instructions about this condition of the experiment were clear and I understood them.

   1 2 3 4 5

2. Condition was mentally tiring.

   1 2 3 4 5

3. Condition was physically tiring.

   1 2 3 4 5

4. I was able to remain focused until the end of each run.

   1 2 3 4 5

5. In this condition, I found it easy to concentrate on imagining movement of my hand as the hand on the screen moved.

   1 2 3 4 5

Following questions are related to the condition of the experiment in which **vibrotactile guidance was present**. Please, read the questions carefully and answer each question by circling one number from 1 to 5. (1: Strongly disagree, 2: Disagree, 3: Neither agree nor disagree, 4: Agree, 5: Strongly agree)

1. Instructions about this condition of the experiment were clear and I understood them.

   1 2 3 4 5

2. Condition was mentally tiring.

   1 2 3 4 5

3. Condition was physically tiring.

   1 2 3 4 5

4. I was able to remain focused until the end of each run.

   1 2 3 4 5

5. In this condition, I found it easy to concentrate on imagining movement of my hand as the hand on the screen moved (while at the same time concentrating on the vibrotactile guidance).

   1 2 3 4 5

6. In this condition, I found it easy to concentrate on vibrotactile guidance and detect its direction.

   1 2 3 4 5

7. I think I detected and reported most of the mismatched trials (trials in which visual guidance was not in the same direction as vibrotactile guidance) correctly.

   1 2 3 4 5
