# Supplementary material for: Directional Decoding From EEG in a Center-Out Motor Imagery Task With Visual and Vibrotactile Guidance
Source: Front Hum Neurosci. 2021 Sep 24;15:687252. doi: 10.3389/fnhum.2021.687252 (PMC8497713; doi:10.3389/fnhum.2021.687252)
Supplement: Supplementary file 2 [file Data_Sheet_2.PDF]

Supplementary Material

1 SUPPLEMENTARY DOCUMENTS

The questionnaire referenced in the manuscript is attached as a separate file.

2 SUPPLEMENTARY TABLES AND FIGURES

Figure S1 depicts the EEG sensor layout.

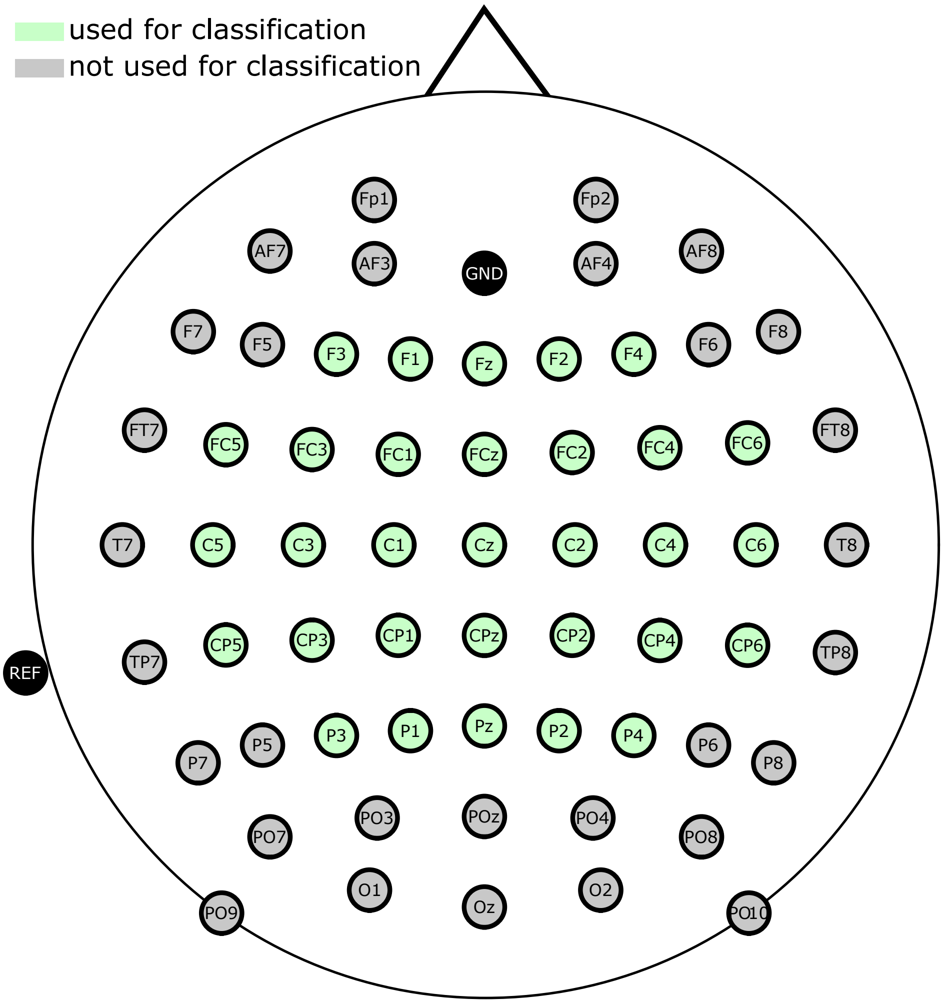

Figure S1. EEG electrode layout. Electrodes shaded in green were used for classification, while electrodes shaded in grey were included for artifact removal, but omitted for classification.

Table S1. Number of trials falsely identified as incongruent, and number of incongruent trials identified correctly.

Participant	False positives (out of 120)	Correct (out of 12)
S1	0	8
S2	0	12
S3	3	9
S4	0	6
S5	0	11
S6	0	12
S7	0	11
S8	0	10
S9	0	9
S10	4	12
S11	0	12
S12	1	8
S13	0	12
S14	0	11
S15	0	8
avg	0.53	10.07
stdev	1.25	1.94

Table S1 lists the number of false positives and true positives for the subjective detection of incongruent trials.

Figure S2 shows the accuracies for the classification between directions based on spectral features.

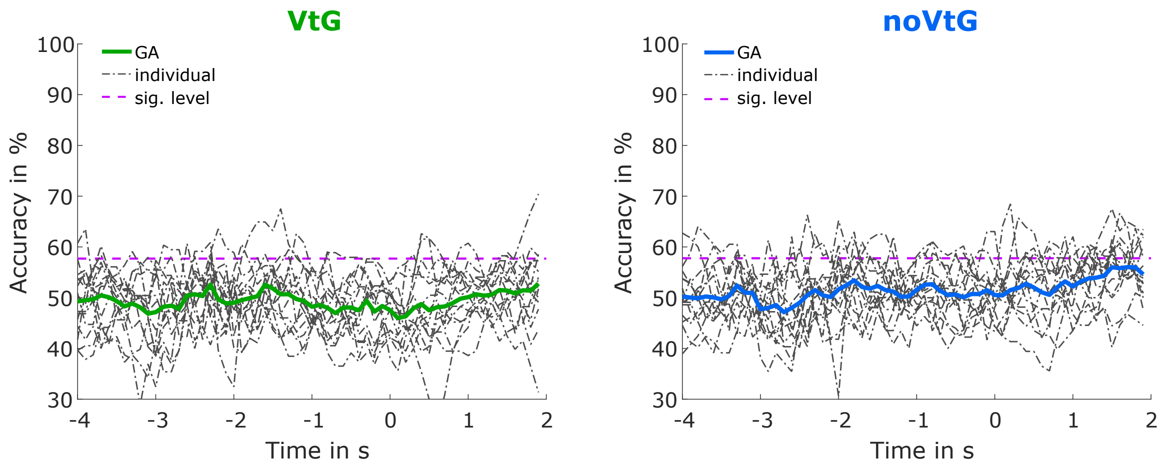

Figure S2. Classification results for direction right vs. direction up, based on spectral features. The grand-average accuracies are depicted as thick solid lines, in green for condition VtG, and in blue for condition noVtG. Single-subject accuracies are represented by dash-dotted dark grey lines.

Table S2 contains the (peak) accuracies of all participants. The peak accuracies for classification between directions based on spectral features are not included. As is evident from figure S2, accuracies are around chance level in this case, and therefore presenting peak accuracies would not be informative.

Table S3 shows a summary of participants' answers to the questions on the questionnaire which were related to perceived effort. Two participants did not fill out the questionnaire correctly (one fully, one partly).

Table S2. Overview of classification accuracies.

Participant	Directions peak acc. in %		MI vs. baseline acc. in %				Conditions peak acc. in %	
	amplitude		amplitude		spectral		amplitude	spectral
	VtG	noVtG	VtG	noVtG	VtG	noVtG		
S1	70.94	64.35	78.87	72.52	79.91	86.09	78.62	72.41
S2	73.87	69.30	80.46	76.40	95.05	91.67	86.49	81.33
S3	70.91	75.70	89.45	91.50	82.73	70.56	74.73	73.73
S4	59.65	58.18	81.93	77.18	73.25	63.64	76.15	71.47
S5	67.54	60.53	86.06	82.12	85.53	78.51	84.82	74.14
S6	58.41	65.52	75.69	59.60	84.96	67.24	76.25	79.06
S7	76.36	72.22	82.09	83.05	88.18	81.48	82.84	68.88
S8	61.68	67.59	82.33	83.05	89.72	86.57	85.22	74.42
S9	74.53	66.36	93.11	88.18	85.44	70.00	90.01	66.21
S10	73.04	66.99	82.96	90.78	82.61	79.41	78.36	68.64
S11	74.77	75.00	84.13	74.43	79.44	73.91	73.99	77.35
S12	80.00	66.39	76.35	74.85	79.28	76.47	70.43	73.91
S13	60.58	66.67	82.89	84.36	78.85	73.61	82.45	69.34
S14	69.72	65.71	82.64	83.52	86.49	75.00	82.15	75.81
S15	73.11	64.66	88.34	70.23	68.22	58.85	83.31	68.83
avg	69.67	67.01	83.15	79.49	82.64	75.53	80.39	73.04

Table S3. Summary of subjective ratings on the questionnaire. The top rows show the ratings regarding how mentally, and physically tiring the task was perceived to be, respectively, where a low rating indicates that it was not tiring, and conversely, a high rating indicates that it was very tiring. The bottom rows show the ratings for how easy participants found it to remain focused throughout the experiment, to concentrate on the MI task, and for condition VtG additionally how easy they found it to concentrate on the vibrotactile guidance. The columns labelled 1-5 (for each condition) contain the number of participants that gave the respective rating on the questionnaire. The columns labelled with a question mark indicate the number of participants that did not fill out the questionnaire correctly, and the rightmost column for each condition shows the average rating for each question. The two rightmost columns of the table indicate the numbers of participants that individually provided a higher rating to the respective questions in either condition.

	VtG							noVtG							VtG higher	noVtG higher
Likert scale rating	1	2	3	4	5	?	avg	1	2	3	4	5	?	avg		
mentally tiring	2	2	2	5	2	2	3.2	2	4	1	6	1	1	3.0	3	1
physically tiring	8	2	2	1	0	2	1.7	7	3	1	3	0	1	2.0	0	3
able to remain focused	0	3	1	6	3	2	3.7	0	4	3	4	3	1	3.4	2	1
easy to concentrate on MI	0	2	4	3	4	2	3.7	0	1	2	5	6	1	4.1	3	5
easy to concentrate on VtG	0	1	2	2	8	2	4.3	-	-	-	-	-	-	-	-	-

The missing answers are indicated by question marks. In the top two questions presented in table S3, a high rating indicated a high effort (very tiring), while in the bottom three questions, a high rating indicated a low effort (easy to remain focused/concentrate).
